# Granulomatosis With Polyangiitis as an Etiology of Silent Sinus Syndrome: A Case Report

**DOI:** 10.7759/cureus.61442

**Published:** 2024-05-31

**Authors:** Nicholas Kramer, Brandon Manthei, Luke Speier, Jo-Lawrence M Bigcas, Scott Manthei

**Affiliations:** 1 College of Osteopathic Medicine, Touro University Nevada, Henderson, USA; 2 Department of Otolaryngology-Head and Neck Surgery, Kerkorian School of Medicine at the University of Nevada Las Vegas, Las Vegas, USA; 3 Department of Otolaryngology, Nevada Ear and Sinus Institute, Las Vegas, USA

**Keywords:** c-anca, anca associated vasculitis, functional endoscopic sinus surgery, ent procedures, granulomatosis with polyangiitis (gpa), wegeners granulomatosis, silent sinus syndrome

## Abstract

Silent sinus syndrome (SSS) is a rare condition characterized by the collapse of the maxillary sinus and the sinking of the eye socket (enophthalmos). Only around 100 cases of SSS have been reported so far. The underlying cause of this condition is the chronic obstruction of the osteomeatal complex, which leads to sinus contraction. In this case, we present a novel finding linking SSS with granulomatosis with polyangiitis (GPA). The patient described is a 39-year-old male who was diagnosed with SSS after a prolonged period of sinus pressure, headaches, epistaxis, and generalized congestion. Additionally, the patient reported a significant autoimmune history, including a previous occurrence of ANCA-mediated glomerulonephritis. Surgical intervention revealed the presence of significant granulation tissue, while histopathological examination identified areas of necrosis, vasculitis, and multinucleated giant cells consistent with GPA. This finding was further supported by the detection of positive blood c-ANCA. This case is particularly noteworthy as it is the first reported instance of GPA causing SSS. It serves as an excellent example to illustrate the underlying pathophysiology of SSS.

## Introduction

Silent sinus syndrome (SSS) is defined as the combination of maxillary atelectasia and evidence of enophthalmos. [1] SSS is a seemingly rare condition within the literature, with around 100 cases described. [2] The proposed pathophysiology behind SSS, which will be discussed in more detail later, involves chronic obstruction of the osteomeatal complex and subsequent contraction of the maxillary sinus. [3] Considering this mechanism, the case provides an interesting casual relationship between SSS and a case of ANCA-positive granulomatosis with polyangiitis (GPA), formerly known as Wegener’s. It is worth noting that SSS caused by or concurrent with GPA has not been previously represented in the literature, making this a novel case.

## Case presentation

**Figure 1:**
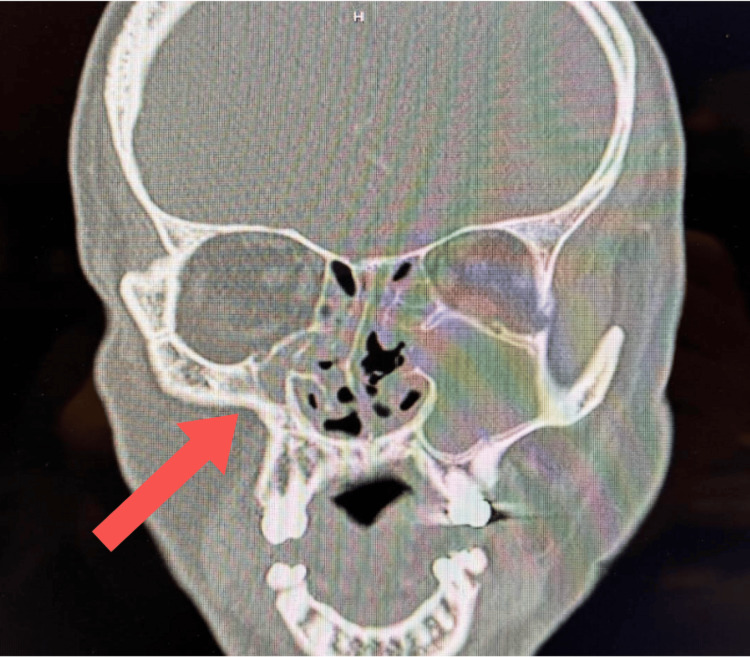
Right maxillary sinus contracture consistent with silent sinus syndrome

A 39-year-old male presented to the clinic with a history of sinus pressure, headaches, epistaxis, and generalized congestion, persisting for three months. Physical examination revealed mucopurulence in the right nasal cavity with bilateral hypertrophic inferior turbinates and dry blood on the left nasal septum. The patient was sent for sinonasal CT and provided a two-week course of clindamycin and prednisone. The CT results indicated severe sinusitis in the ethmoidal air cells bilaterally, left maxillary sinus opacification extending through the left osteomeatal complex, and a small-caliber right maxillary sinus with opacification of the right osteomeatal complex. Furthermore, moderate mucosal thickening was observed in the right frontal sinus, while minor mucosal thickening was noted in the left frontal and sphenoid sinuses. The nasal septum was severely deviated to the right. At his next appointment, the patient was informed of his results (Figure 1), consistent with right maxillary SSS and severe pansinusitis. The physical exam revealed worsening sinonasal obstruction, with the right nasal cavity nearly completely obstructed with mucopurulent discharge and the left 80% obstructed. The patient was informed of the risk of impending cellulitis due to the severity of his sinonasal disease and agreed to undergo surgical treatment pending cardiac clearance for a history of atrial fibrillation treated with ablation. In the interim, the patient was placed on maximal medical therapy.

Unfortunately, cardiac clearance took longer than anticipated, and two weeks later, the patient ended up in the emergency department with right eye swelling and exquisite right-sided sinus tenderness. Emergency department evaluation, including a CT scan, revealed preseptal cellulitis and worsening pansinusitis. In-office follow-up involved continued counseling on the gravity of his condition, emphasis on obtaining cardiac clearance, and instructions to continue therapy and to promptly report to the emergency department if his condition worsened before surgery. Two months later, the patient obtained cardiac clearance, and surgical planning was confirmed. Fortunately, the patient had no other episodes of cellulitis.

**Figure 2:**
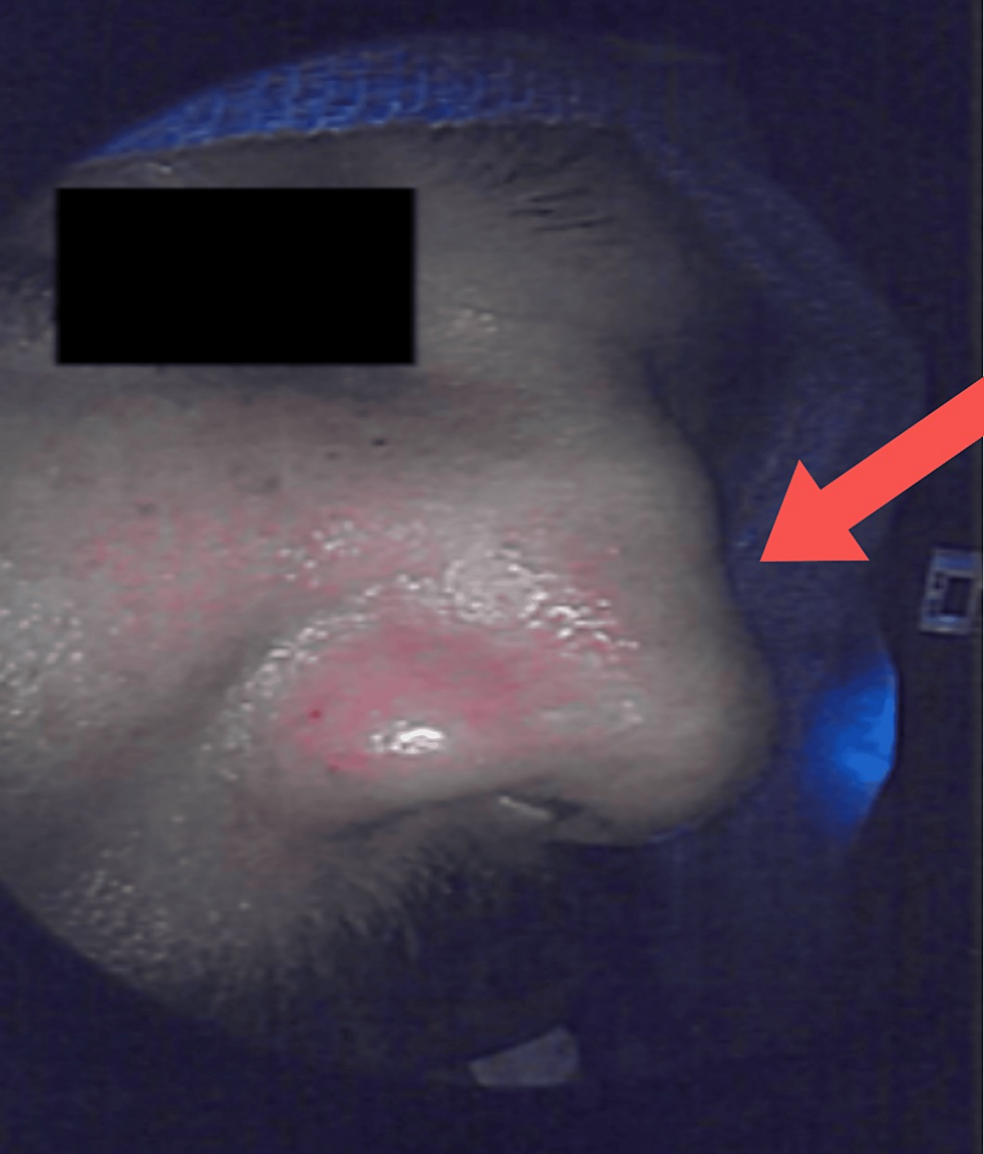
Preoperative view of the saddle nose deformity

During the pre-operative interview on the day of surgery, an unknown mild saddle-nose deformity was discovered (Figure 2). The patient said he noticed his nose gradually caving in for several weeks. The significance of this observation was explained to the patient, emphasizing its potentially ominous nature and that this would be followed with tissue biopsy and pathologic review. Septoplasty, endoscopic sinus surgery with total bilateral ethmoidectomy, and bilateral maxillary sinus debridement were performed.

**Figure 3:**
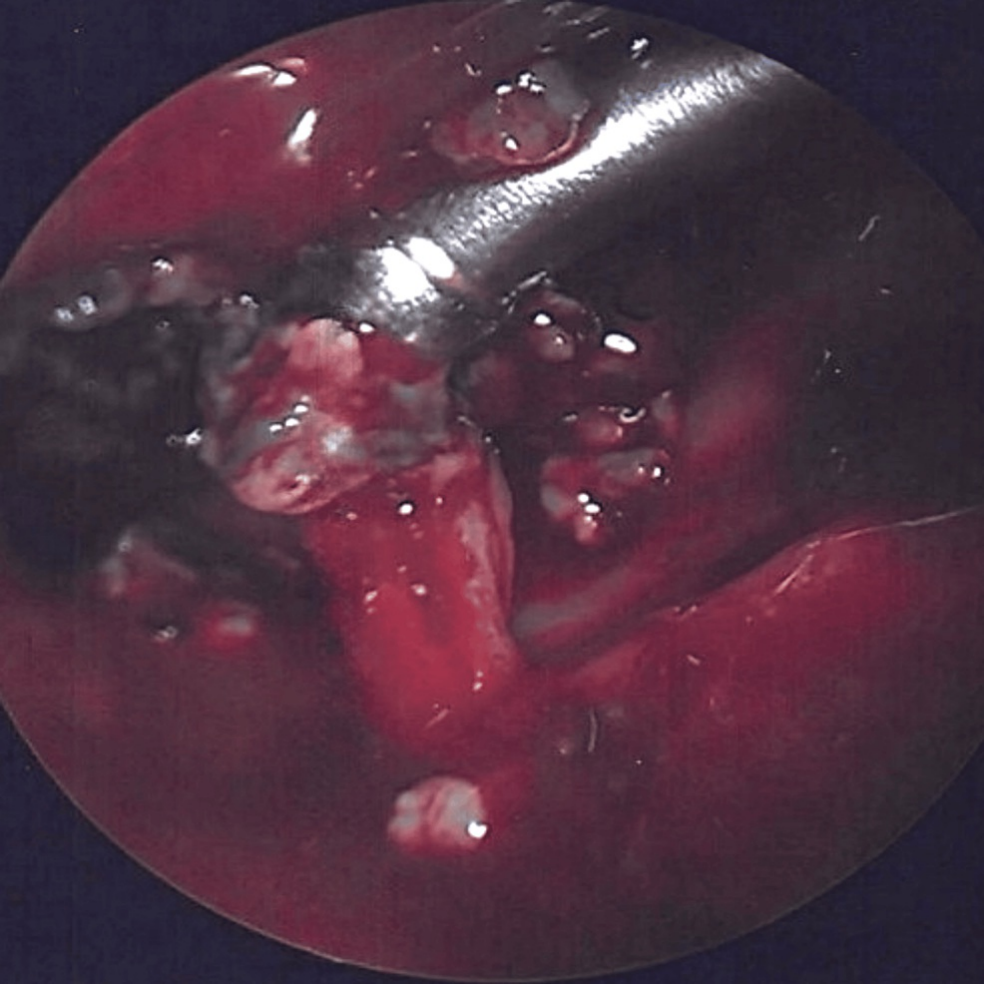
Intraoperative granulation tissue in the right osteomeatal complex

Intraoperatively, remarkable granulation tissue and crusting were observed in both nasal passageways, apparently coming from the septum, almost bubbling out of the septal cartilage, with significant collection in the right osteomeatal complex, as depicted in Figure 3. Additionally, there was adhesion of the middle turbinate on the left side and the inferior turbinate on the right side. Surprisingly, the sinuses appeared quiet, with the right maxillary sinus showing signs of contraction consistent with SSS. Furthermore, elongation of the right orbit, indicative of right maxillary sinus contracture, and thickening of the maxillary bone were noted. Due to the clinical suspicion of a possible vasculitic process and the intraoperative fragility of the septum, septoplasty was performed by external reduction to midline without mucosal incision to reduce the risk of septal perforation. Cultures and tissue samples were obtained for histopathologic review to investigate the condition further.

The patient recovered well from the procedure. The tissue biopsy from the right maxillary sinus mucosa revealed areas of necrosis, vasculitis, and rare multinucleated giant cells consistent with GPA. Furthermore, the patient's blood testing for c-ANCA came back positive along with a high c-ANCA titer (1:80). Elevated levels of C-reactive protein, rheumatoid factor, and sedimentation rate were also observed.

At the two-week follow-up, the patient was seen to be recovering well from the surgery. Due to the intraoperative findings, laboratory studies, and a strong clinical suspicion, we decided to delve deeper into the patient's medical history, with a particular focus on autoimmunity. The patient revealed that in 2020, he had a renal biopsy performed at the hospital for hematuria and poor kidney function. The renal biopsy revealed pauci-immune necrotizing and crescentic glomerulonephritis, consistent with ANCA-associated glomerulonephritis. He was subsequently treated with three courses of rituximab, and his autoimmune condition stabilized in 2022. Unfortunately, during this period, he only received medical attention from nephrology and oncology, with no consultation from rheumatology. Due to the stabilizing of his condition in 2022, it was not mentioned at prior office visits. 

**Figure 4:**
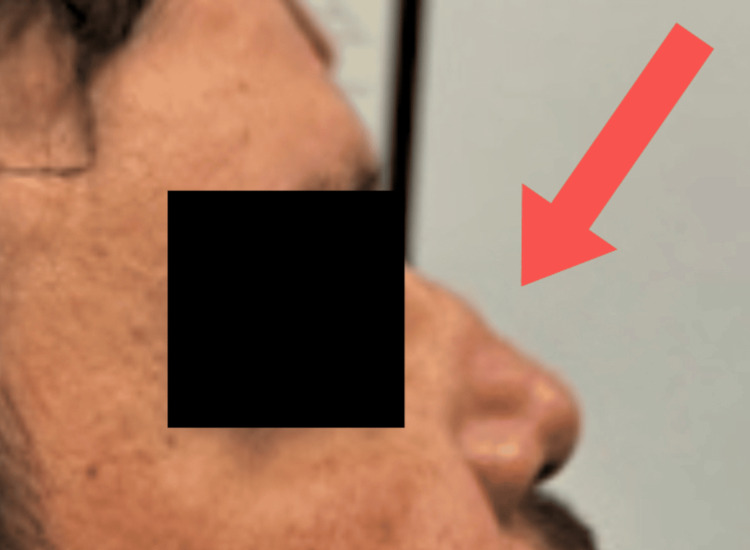
Worsening of the saddle nose deformity

After collecting this history, the patient expressed concern about the increasing collapse of his nose, which was also noted by the clinicians (Figure 4). In addition, the patient reported that his eyes had been tearing significantly over the last week. This new epiphora and worsening of the saddle nose deformity prompted the team to refer the patient to rheumatology and ophthalmology. The patient was informed that further operative intervention, including correction of the saddle nose deformity, will be discussed after his rheumatologic condition stabilizes. Follow-up is currently being maintained.

## Discussion

SSS is a seemingly rare condition. However, due to the lack of studies assessing its prevalence in the general population, it is challenging to determine the true rarity of this disease [2]. Regarding nomenclature, it is important to recognize that SSS is often used interchangeably with chronic maxillary atelectasis (CMA) [1]. However, some have insisted on grouping SSS within the classification system of CMA [4]. SSS typically presents in the fourth decade of life with equal prevalence among the sexes. The primary risk factor for the disease is underlying aberrant nasal anatomy, which is logical considering the proposed pathophysiologic mechanism of infundibular obstruction leading to negative maxillary sinus pressure. Over time, this leads to absorption of the sinus, which causes a gradual indrawing of the walls, including the orbital floor [3,5].

The original definition of SSS from 1994 excluded traumatic and iatrogenic etiologies. However, there has been a shift in recent literature towards including these causes, among others, in the definition of SSS. A significant point of debate lies within symptomatology. In a systematic review conducted by Rosso et al., 28 original articles on SSS were isolated. Of these, nine studies included patients with sinonasal symptoms such as diplopia, sinusitis, rhinorrhea, post-nasal drip, facial pain, and facial pressure. Conversely, ten articles used sinonasal symptoms as exclusion criteria for SSS. Nevertheless, most studies either do not address symptomatology or consider it part of the definition of SSS. The fundamental diagnostic criteria extrapolated from their review are enophthalmos or hypoglobus with contraction of the maxillary sinus identified on CT or MRI, regardless of whether the patient is symptomatic or asymptomatic [1].

The cornerstone of treatment for SSS is functional endoscopic sinus surgery (FESS), usually including uncinectomy and maxillary antrostomy to relieve osteomeatal complex (OMC) obstruction [1,3,5-7]. Rosso et al. found that upon resolution of OMC obstruction with FESS, enophthalmos and orbital floor depression tend to reverse back to normal [1].

GPA is a rare autoimmune disease characterized by small vessel vasculitis with clinical manifestations of necrotizing vasculitis and granulomatous inflammation, causing multisystem disease. The incidence of GPA is 5-10 cases per million and typically occurs in those between 45 and 60 years old with no predilection toward either sex [8,9].

The etiology and pathogenesis of GPA remain poorly understood. The disease appears to arise from a combination of environmental and individual genetic risk factors. There is compelling evidence suggesting an association between the development of the disease and the HLA-DPB1*0401 and HLA-DPB4. Environmental factors associated with the development of GPA include infections, exposure to toxic inhalants, and various medications, including antithyroid, anticancer, and anti-infectious drugs. Among infectious associations, patients with GPA have a higher carrying rate of sinonasal Staphylococcus aureus than non-diseased individuals [10].

In approximately 85% of patients with GPA, the first symptoms include sinonasal involvement. This commonly manifests as nasal obstruction and may involve paranasal sinus damage. The sinonasal manifestations typically originate from vasculitic damage to Kisselbach's plexus in the nasal septum, which may result in a saddle nose deformity [11]. Additional manifestations of the disease include ophthalmologic involvement. Up to 58% of individuals with GPA experience ocular manifestations, including conditions like scleritis, dacryocystitis, and proptosis [12].

Various diagnostic criteria have been proposed for GPA, and the disease manifests with a range of severity levels and affected organ systems, which complicates the diagnostic process. [13,14] The 2022 American College of Rheumatology/European Alliance of Associations for Rheumatology classification lays out succinct point-based criteria for granulomatosis with polyangiitis. As outlined by the college:

"The final criteria and their weights were as follows: bloody nasal discharge, nasal crusting or sino-nasal congestion (+3); cartilaginous involvement (+2); conductive or sensorineural hearing loss (+1); cytoplasmic antineutrophil cytoplasmic antibody (ANCA) or anti-proteinase 3 ANCA positivity (+5); pulmonary nodules, mass or cavitation on chest imaging (+2); granuloma or giant cells on biopsy (+2); inflammation or consolidation of the nasal/paranasal sinuses on imaging (+1); pauci-immune glomerulonephritis (+1); perinuclear ANCA or antimyeloperoxidase ANCA positivity (-1); and eosinophil count ≥1×109 /L (-4). After excluding mimics of vasculitis, a patient with a diagnosis of small- or medium-vessel vasculitis could be classified as having GPA if the cumulative score was ≥5 points. When these criteria were tested in the validation data set, the sensitivity was 93% (95% CI 87% to 96%) and the specificity was 94% (95% CI 89% to 97%)" [15]. 

Based on the given criteria, the presented patient scores (+3) for bloody nasal discharge and sinonasal congestion, (+2) for cartilaginous involvement (septal cartilage), (+5) for c-ANCA positivity, (+2) for giant cells on biopsy, (+1) for consolidation of the nasal/paranasal sinuses on imaging, and (+1) for history of pauci-immune glomerulonephritis, with no negative scores due to the absence of other antibodies or eosinophilia. Considering the cumulative score of 14, which surpasses the college score cutoff of 5, it is highly reasonable to conclude that this patient has a genuine case of GPA.

The presence of both SSS and GPA in this patient raises the question of whether GPA is the true cause of the SSS. To address this, we again reference the pathophysiology of SSS: infundibular obstruction leading to negative maxillary sinus pressure [3]. Tateyama et al. report the most common findings of sinonasal GPA being granulation tissue (52.9%), crusting (47.1%), redness/erosion (29.4%), necrosis (23.5%), purulent drainage (23.5%), saddle nose (17.6%), loss of turbinate (17.6%), and nasal septal perforation (17.6%) [16]. The patient exhibited most of these findings. Given the lack of other identifiable causes of infundibular obstruction and the consistency of intraoperative findings with Tateyama et al.'s GPA sinonasal findings, it is reasonable to conclude that this is truly a case of GPA causing SSS.

## Conclusions

This case presents a compelling account of the development of SSS from GPA as a novel etiology. These two conditions are infrequently discussed in the existing literature, and SSS arising from GPA has not been documented. Additional recordings of the concurrence of these diseases will benefit multiple physician subspecialties in recognizing SSS as a potential complication of GPA. Ongoing patient monitoring and collaboration with a multidisciplinary team of specialists will be maintained to ensure comprehensive care.
